# Population Genetic Structure of the Endangered Long-Tailed Goral (*Naemorhedus caudatus*) in South Korea Revealed by Genome-Wide Markers from a 3-RADseq Approach

**DOI:** 10.3390/ani16142189

**Published:** 2026-07-14

**Authors:** Donggul Woo, Ju-Won Hwang, Gyeong-Min Lee, Younha Han, Yeong-Seok Jo

**Affiliations:** 1 Endangered Species Recovery Center, National Institute of Ecology, Yeongyang 36531, Republic of Korea; martes@nie.re.kr (D.W.); gorani@nie.re.kr (Y.H.); 2 Department of Biology Education, Daegu University, Gyeongsan 38453, Republic of Korea; oklhy1230@naver.com (J.-W.H.); lgm0109@naver.com (G.-M.L.)

**Keywords:** *Naemorhedus caudatus*, 3-RADseq, population genomics, genetic isolation, landscape fragmentation, endangered species, conservation management

## Abstract

The long-tailed goral (*Naemorhedus caudatus*) is facing severe habitat fragmentation along the Baekdudaegan Mountain Range, South Korea, due to expanding anthropogenic infrastructures and ASF fence installations. To evaluate the evolutionary impacts of these barriers, we assessed the genome-wide genetic diversity and structural connectivity of South Korean goral populations using a high-resolution triple-digest restriction site-associated DNA sequencing (3-RADseq) framework. Out of 36 biological samples collected across regional demes, 34 individuals were successfully genotyped, yielding an initial variant pool of 68,041 raw single nucleotide polymorphisms (SNPs) that filtered down to 9908 high-quality independent SNPs. Bayesian model clustering and principal coordinate analysis consistently identified K = 2 as the optimal number of genetic clusters, resolving a deep, distinct genetic split between the continuous Northern and Southern populations. This pronounced genetic structuring indicates severely restricted contemporary gene flow, heightening vulnerability to genetic drift and inbreeding depression within isolated terminal patches. Our genomic evidence suggests that single-protected-area approaches are insufficient; effective conservation priorities must focus on restoring landscape continuity and ecological corridors to revive historical migration pathways and preserve the adaptive potential of this endangered mountain ungulate.

## Simple Summary

The long-tailed goral is a highly threatened wild mountain mammal protected in South Korea. Due to increasing human activities, highway networks, and continuous protective African Swine Fever fencing installations, wild animal habitats along major mountains have become severely cut off from one another. To evaluate how these divisions affect the genetic health of remaining animals, we tracked thousands of DNA markers across wild individuals using an advanced high-throughput sequencing method. Our genomic analysis discovered that the wild goral populations are deeply fractured into two primary genetic lineages: a continuous northern block and a severely isolated southern boundary group. This genetic isolation poses a potential threat to their long-term survival by increasing susceptibility to inbreeding and the loss of genetic diversity, particularly following severe natural mortality shocks. These findings demonstrate that saving this endangered species requires building continuous landscape bridges and protective ecological paths to re-establish historical migration networks across the country.

## 1. Introduction

**Figure 1:**
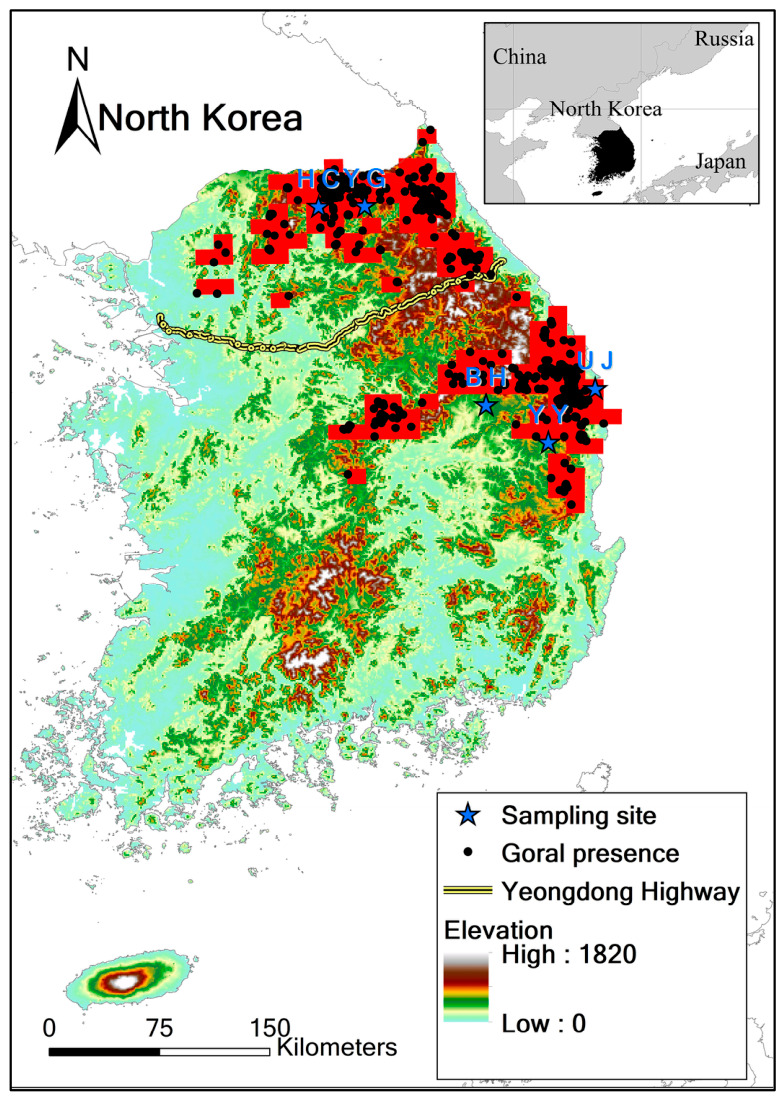
Distributional map of the long-tailed goral (*Naemorhedus caudatus*) in South Korea derived from surveys during 2019–2022. Red squares with black dots denote the exact grids and localities where goral presence has been empirically confirmed. The background terrain is constructed using a digital elevation model (DEM). See Table 1 for population abbreviations.

The long-tailed goral (*Naemorhedus caudatus*) is a specialized mountain ungulate primarily distributed across East Asia, inhabiting rugged, high-altitude rocky terrains [1]. In South Korea, the species is designated as an endangered wildlife species (Class I) by the Ministry of Environment and a Natural Monument (#217) due to severe population declines driven by historical overexploitation, poaching, and continuous habitat loss [2]. Geographically, the goral populations in South Korea exhibit a discontinuous and clumped distribution pattern along the major ridges of the Baekdudaegan Mountain Range, including the Demilitarized Zone (DMZ), Seorak-san, and the Uljin-Samcheok regions (Figure 1) [3]. However, recent anthropogenic landscape alterations—such as transport infrastructure expansion and the extensive installations of fences to mitigate African Swine Fever (ASF)—have further compounded natural topographical barriers, exacerbating habitat fragmentation and isolating local demes [4].

**Table 1:** Summary of populations and within-population genetic diversity estimates across five populations of *Naemorhedus caudatus* in South Korea. *Na*, mean number of alleles; *Ho*, observed heterozygosity; *H_E_*, expected heterozygosity; *F_IS_*, inbreeding coefficient; SE, standard error.

Population (Acronym)	Number of Samples	*Na* [SE]	*Ho* [SE]	*H_E_* [SE]	*F_IS_* [SE]
Bonghwa (BH)	5	1.749 [0.005]	0.285 [0.003]	0.266 [0.002]	0.074 [0.005]
Hwacheon (HC)	9	1.845 [0.004]	0.299 [0.002]	0.288 [0.002]	−0.041 [0.004]
Uljin (UJ)	6	1.773 [0.005]	0.323 [0.003]	0.273 [0.002]	−0.161 [0.004]
Yanggu (YG)	10	1.910 [0.003]	0.310 [0.002]	0.304 [0.002]	−0.020 [0.003]
Yeongyang (YY)	4	1.709 [0.005]	0.294 [0.003]	0.259 [0.002]	−0.134 [0.005]

Anthropogenic habitat fragmentation and subsequent population isolation pose critical threats to the long-term persistence of endangered mammals by restricting gene flow and accelerating genetic erosion [5]. Small, isolated populations are highly susceptible to genetic drift, severe bottlenecks, and inbreeding depression, which ultimately diminish their adaptive potential against environmental changes or disease outbreaks [6]. While ecological field surveys using camera traps and footprint tracking from 2019 to 2022 have successfully identified the distributional ranges of long-tailed goral in South Korea [4], these traditional monitoring approaches fail to capture the underlying evolutionary processes, such as contemporary gene flow, fine-scale genetic structure, and cryptic barriers to movement [7].

Previous genetic assessments of long-tailed goral in South Korea have primarily relied on mitochondrial DNA (mtDNA) sequencing and microsatellite markers [8,9,10]. Specifically, prior mtDNA research focused on phylogenetic relationships utilizing samples exclusively from the northern population [9,10], whereas studies employing microsatellite loci revealed a distinct latitudinal genetic split between northern and southern groups [8,9]. The previous mtDNA and microsatellite markers suffer from inherent constraints, such as low genomic coverage, limited marker counts, and high stochastic variance. Consequently, such low-resolution marker systems often lack the statistical power required to resolve subtle genetic sub-structuring or cryptic genetic fracturing driven by recent anthropogenic landscape fragmentation along the continuous Baekdudaegan mountain range [11,12,13].

To overcome these limitations, next-generation sequencing (NGS)-based restriction site-associated DNA sequencing (RADseq), specifically the triple-digest RADseq (3-RADseq) approach, has emerged as a robust and well-established framework in conservation genomics [14,15]. By utilizing a tailored combination of specific restriction enzymes, 3-RADseq enables the cost-effective, highly reproducible, and high-throughput discovery of thousands of genome-wide single nucleotide polymorphisms (SNPs) across the genome [15]. This approach provides a high-resolution molecular genomics toolkit capable of bypassing the limitations of traditional markers, thereby offering the precise sensitivity required to evaluate contemporary micro-geographic barrier effects, assess population-level genetic health, and identify subtle genetic erosion in endangered alpine species [16,17].

Therefore, the main objectives of this study were to: (1) perform a comprehensive genome-wide SNP analysis of long-tailed goral populations in South Korea using the 3-RADseq framework; (2) determine the fine-scale genetic diversity and spatial genetic structure across regional populations; and (3) assess genetic differentiation and connectivity to identify isolated management units requiring targeted conservation prioritization and ecological corridor restoration.

## 2. Materials and Methods

### 2.1. Sample Collection and DNA Extraction

Wild goral specimens, primarily consisting of high-quality tissue and hair matrices, were officially provided by the Genetic Bank of the Endangered Species Center at the National Institute of Ecology (NIE), South Korea, for genomic analysis. A total of 36 samples of *Naemorhedus caudatus* were collected across South Korea, representing major regional populations along the latitudinal gradient of the Baekdudaegan Mountain Range, including Yanggu (YG), Hwacheon (HC), Uljin (UJ), Bonghwa (BH) and Yeongyang (YY). To ensure optimal downstream library preparation, genomic DNA was extracted using commercial DNA isolation kits (Qiagen, Valencia, CA, USA) following the manufacturers’ protocols. The quantity and purity of the extracted DNA were strictly evaluated using a Qubit Fluorometer and agarose gel electrophoresis (Thermo Fisher Scientific, Waltham, MA, USA), ensuring a standardized starting concentration of ≥20 ng/μL for all samples [17].

### 2.2. 3-RADseq Library Preparation and Sequencing

The 3-RADseq libraries were prepared by modifying the standardized triple-digest protocol to optimize fragment size selection for the long-tailed goral genome [15]. Genomic DNA was digested using a precise enzyme combination consisting of EcoRI-HF and XbaI to generate targeted genomic fragments, while NheI was simultaneously applied to cleave dimerized adapters [17]. Digestion and adapter ligation were executed simultaneously in a total reaction volume of 15 μL, containing 10 μL of template DNA, 1 μL of each specific double-stranded adapter, and 3 μL of the restriction enzyme mix [17]. The mixture was incubated in a thermal cycler at 37 °C for 15 min, followed by enzyme inactivation at 80 °C for 15 min [17]. Subsequently, T4 DNA ligase mix was introduced to cultivate permanent adapter ligation at 16 °C for 6 h.

To confirm successful adapter integration, a test polymerase chain reaction (PCR) amplification was performed in a 20 μL reaction containing Bioneer multiplex premix alongside iTru5_test and iTru7_test primers (Bioneer Corp, Daejeon, Republic of Korea) [16]. The thermal cycling conditions comprised an initial denaturation at 95 °C for 10 min, followed by 30 cycles of 95 °C for 30 s, 60 °C for 30 s, and 72 °C for 45 s, with a final extension at 72 °C for 5 min [17]. Validated libraries were pooled, and size selection was executed to capture fragments ranging between 400 and 500 bp. The finalized 3-RADseq libraries were sequenced on an Illumina HiSeq X-10 platform (Illumina, San Diego, CA, USA) to generate high-throughput paired-end reads.

### 2.3. Bioinformatic Processing and SNP Filtering

Raw sequence reads were demultiplexed, checked for quality, and processed utilizing the pipeline implemented in Stacks v.2.41. Reads exhibiting low Phred quality scores or ambiguous barcode sequences were discarded. Secondary automated filtering selected variant sites present in at least 80% of individual samples across all target populations [17]. To eliminate potential linkage disequilibrium and ensure independent data structures, only the primary SNP per RAD locus was retained for downstream analysis [16]. Strict population genomic quality controls were enforced using PLINK v.1.9 and Tassel 5.0 to exclude loci deviating significantly from Hardy–Weinberg equilibrium (*p* < 10 × 10^−6^), genotypes exhibiting >30% missing data, and rare variants possessing a minor allele frequency (MAF) ≤ 0.05, yielding a finalized pool of 9908 robust genome-wide SNPs [17].

We acknowledge that the sample sizes across the five designated demes are relatively small and uneven (BH = 5, HC = 9, UJ = 6, YG = 10, and YY = 4), which represents an inherent challenge when sampling highly endangered and elusive alpine mammals. However, by implementing these exceptionally stringent quality control thresholds and bioinformatic filtering, we effectively minimized stochastic noise, allele calling errors, and potential sampling biases. This rigorous framework ensures the high accuracy and genomic robustness of the 9908 finalized SNPs, thereby providing sufficient statistical power to resolve fine-scale population structure and cryptic genetic fracturing without over-parameterization.

### 2.4. Population Genetic and Statistical Analyses

Genome-wide genetic diversity metrics—including the mean number of alleles (*Na*), observed heterozygosity (*Ho*), expected heterozygosity (*H_E_*), and inbreeding coefficient (*F_IS_*)—were computed for each regional deme using GenAlEx 6.5 [16,17]. Spatial genetic structure and individual cluster assignments were resolved using the Bayesian model-based approach in STRUCTURE v.2.3.4 [18]. The analysis was executed using an admixture model with correlated allele frequencies, setting the number of hypothetical clusters (K) from 1 to 7 with 10 independent replicates per K. Structure Harvester was used to determine the optimal K value via the Evanno ΔK method [19]. To visually corroborate the cluster boundaries, a Principal Coordinate Analysis (PCoA) based on Nei’s genetic distance was performed using GenAlEx 6.5 [20]. Finally, contemporary genetic differentiation and pairwise fixation indices (*F_ST_*) between the designated regional populations were calculated using Arlequin v.3.5 to quantify the degree of genetic isolation and directional gene flow across the landscape [21]. To avoid multiple-comparison inflation, individual SNPs were collapsed into an overall multilocus genotype matrix per individual for global permutation testing (1000 permutations) rather than analyzed via independent locus-by-locus tests. We also conducted a Mantel Test to examine Isolation by Distance (IBD) using the pairwise genetic distances (Slatkin’s linearized *F_ST_* = *F_ST_*/(1 − *F_ST_*)) and log-transformed Euclidean distances for all population pairs in GenAlEx 6.5 [20].

## 3. Results

### 3.1. 3-RADseq Data and SNP Filtering

Through the high-throughput 3-RADseq analysis of long-tailed goral, 36 biological samples were initially processed, among which 34 samples were successfully genotyped and retained for downstream genomic analyses. The initial variant calling across the 34 validated individuals resolved a total of 68,041 raw SNPs. After processing raw sequence reads in Stacks v.2.41, automated filters were enforced to retain loci present in ≥80% of samples across at least five populations, selecting only the first SNP per locus to strictly prevent linkage disequilibrium. Following the stringent population genomic quality control and filtering thresholds specified in the methods section, a finalized core panel of 9908 high-quality genome-wide SNPs was successfully retained for downstream analyses (Supplementary Table S1).

### 3.2. Genome-Wide Genetic Diversity of Regional Populations

The genome-wide genetic diversity parameters computed for the designated sample groupings—categorized under the regional codes BH, HC, UJ, YG, and YY—exposed prominent spatial variations across the evaluated 9908 SNP loci (Table 1). The mean number of alleles (*Na*), observed heterozygosity (*Ho*), expected heterozygosity (*H_E_*) and inbreeding coefficient (*F_IS_*) varied nominally across the evaluated 9908 SNP loci (Table 1). Genetic diversity metrics remained relatively uniform within continuous mountain regions but demonstrated a lower numerical trend near the peripheral limits of the distribution. The detailed population-specific multilocus genetic diversity statistics are summarized in Table 1.

### 3.3. Population Genetic Structure and PCoA

**Figure 2:**
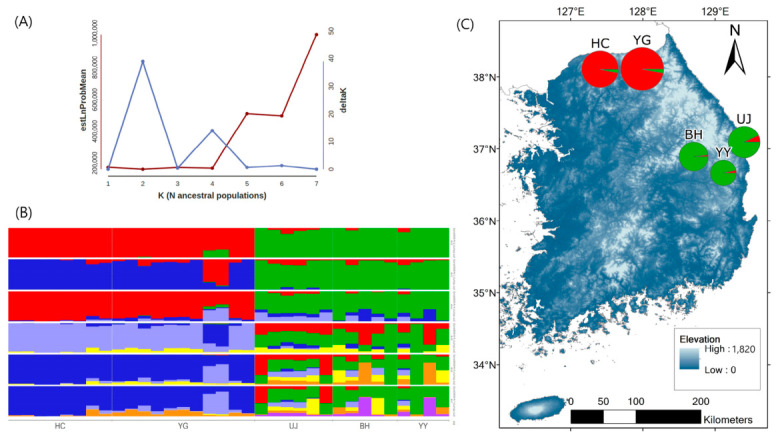
Population genomic structure of *Naemorhedus caudatus* in South Korea. (**A**) Plot of delta K estimated, following Evanno et al. (2005) [19], to determine the optimal K. (**B**) Barplot visualizes group assignments for 34 genotypes from K = 2 (top) to K = 7 (bottom). (**C**) Map of *N. caudatus* sampling sites in South Korea. Pie charts summarized the clustering assignments of individual genotypes within each population (pie size indicate relative sample size). See Table 1 for population abbreviations.

The Bayesian model-based clustering analysis executed via STRUCTURE v.2.3.4 for K = 1–7 was utilized to evaluate the spatial genetic architecture of the 34 goral individuals. Based on the Evanno ΔK method, Structure Harvester identified K = 2 as the optimal number of genetic clusters, revealing a primary genetic split within the South Korean populations (Figure 2A).

At K = 2, the admixture proportions partitioned the 34 individuals into two major latitudinal genetic lineages (Figure 2B,C). Individual barcodes representing the Northern group (HC, YG) and the Southern group (BH, YY, UJ) are clearly divided.

**Figure 3:**
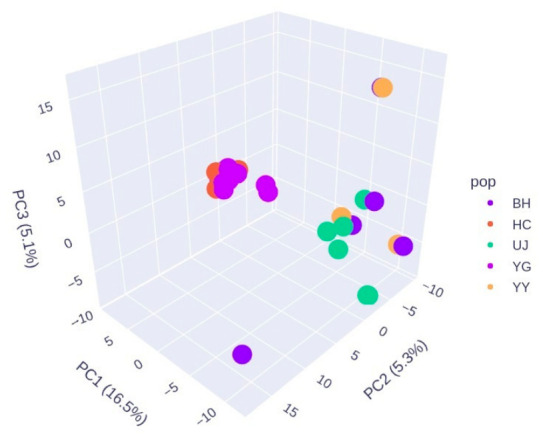
A plot of principal coordinate analysis for 34 *Naemorhedus caudatus* genotypes in South Korea. The first three variance components are plotted. See Table 1 for population abbreviations.

The Principal Coordinate Analysis (PCoA) based on Nei’s genetic distance firmly corroborated the major genetic divergence identified at K = 2. On the three-dimensional coordinate space, the 34 samples were clustered into two groups (Figure 3). The Northern group formed a dense, independent cluster on the left quadrant, while the southern populations occupied separate intermediate positions.

### 3.4. Population Differentiation (F_ST_)

Pairwise fixation indices (*F_ST_*) calculated via Arlequin v.3.5 quantified the degree of genetic differentiation among the regional sample groupings. Reflecting the patterns resolved in the STRUCTURE and PCoA spaces, the pairwise genetic differentiation coefficient (*F_ST_*) varied among the sampled populations, ranging from 0.000 to 0.178 (Table 2). The lowest *F_ST_* values occurred within the southern group (BH, YY, and UJ) and the northern group (YG and HC), reflecting robust intra-regional gene flow despite the clear latitudinal divergence. The complete matrix of pairwise *F_ST_* estimates and associated significance levels (*p*-values) are detailed in Table 2. A Mantel test showed a positive correlation between the genetic differentiations (*F_ST_*) and the linearized geographic distances with significant statistical support (*r* = 0.95, *p* = 0.022).

## 4. Discussion

We acknowledge that our localized sample sizes were relatively small and uneven because of the logistical and legal constraints associated with sampling an endangered species. Such limitations may have reduced the probability of detecting rare alleles and influenced estimates of within-population diversity. Nevertheless, the consistency of the observed north–south differentiation across multiple independent analyses (STRUCTURE, PCoA, and pairwise *F_ST_*), together with the large number of genome-wide SNPs, suggests that the broad-scale population structure identified here is robust [21], although additional sampling would further strengthen these conclusions.

The genome-wide SNP analysis utilizing the high-resolution 3-RADseq framework provided robust, empirical evidence regarding the population structure and genetic isolation of the long-tailed goral (*Naemorhedus caudatus*) in South Korea. The South Korean goral population is optimally partitioned into two distinct genetic lineages, structured along a latitudinal axis across the Baekdudaegan Mountain Range. The contrast in *F_ST_* values observed in this study provides profound insights into this contemporary micro-geographic differentiation [22]. While genetic differentiation within the Northern and Southern clusters remains remarkably negligible, the between-cluster differentiation is exceptionally high. Together with the significant IBD pattern detected by the Mantel test, these results suggest that geographic distance contributes to the observed genetic structure, while the pronounced north–south clustering indicates that additional processes may also have shaped regional differentiation [4,23,24].

While our formal Mantel test did reveal a significant baseline pattern of IBD across the study area (*r* = 0.95, *p* = 0.022), this geographic effect is heavily compounded by natural and anthropogenic landscape features [23,24]. In particular, infrastructure and ASF mitigation fences appear to have rapidly accelerated connectivity loss along the mountain axis [25]. For example, the Yeongdong express highway has bisected this region since the 1970s. While its modernized sections incorporate extensive tunneling, the original route remains fully active today as an open surface national highway running directly parallel to it. The combination with more recent fencing could have reinforced existing barriers to dispersal. However, evaluating the relative contributions of these landscape features will require explicit landscape genetic analyses [22].

Within this framework, the distinct segregation observed in both the STRUCTURE and PCoA analyses is consistent with restricted contemporary gene flow between the northern and southern populations. Although anthropogenic barriers may have reinforced this differentiation [2,4], additional landscape genetics analyses incorporating environmental resistance surfaces will be necessary to quantify their relative contributions. Potential contributing factors include the extensive transportation network and ASF fences deployed for mitigation, which have rapidly choked off historical gene flow across the mid-Baekdudaegan axis [25]. This intensifies the isolation of regional groups, mimicking the island-like severe fragmentation effects previously characterized by high-density genomic markers in insular invasive rodents [17]. In mountain ungulates, continuous gene flow along mountain ridges is vital to maintain genetic diversity and adaptive potential [23,24]; however, these artificial barriers have forced the Southern lineage into severe structural isolation from the northern group [26]. Our genomic data indicate that both the Northern and Southern clusters exhibit relatively high intra-cluster genetic continuity within their respective regions, yet they remain distinctly isolated from each other. This sharp latitudinal fracturing aligns with broader macrogeographic patterns reported in past microsatellite assessments, which highlighted the historical integrity of the northern core populations [8].

This structural isolation signals an acute vulnerability to genetic drift, which compromises the long-term genetic viability of these fragmented southern populations by elevating inbreeding coefficients (*F_IS_*) and reducing heterozygosity (*H_E_*) [27,28]. Encouragingly, our current empirical data indicate that contemporary *F_IS_* values across most sites remain near-zero to negative (ranging from −0.161 to 0.074, Table 1), suggesting that severe inbreeding depression has not yet actively manifested within these localized demes despite their structural isolation. The severe consequences of such genetic erosion are critically underscored when local demes undergo sudden ecological shocks. Although suitable habitats may cross the unsampled intermediate zones along the mountain axis, potential barriers to functional connectivity could exacerbate demographic vulnerabilities. For instance, during the catastrophic winter mass mortality event of 2023–2024 around the Seorak-san region, where over a thousand individuals perished, such restricted connectivity could theoretically impede natural immigration and genetic rescue mechanisms [26,29], highlighting the long-term risk of structural isolation during sudden demographic shocks. Although our neutral SNP panel does not directly evaluate loci associated with local adaptation or specific deleterious traits, the observed structural isolation highlights a heightened demographic and evolutionary vulnerability, suggesting that terminal demes may face diminished long-term capacity to withstand sudden climate-driven crises without the buffering effects of continuous gene flow.

From a conservation genetics perspective, these genomic findings bear immediate, practical implications, proving that traditional, single-protected-area approaches are largely inadequate to secure the evolutionary potential of the long-tailed goral [30]. Based on the deep genetic fracturing, we strongly advocate for the formal designation of the Northern and Southern lineages as two distinct Management Units (MUs). While managing these clusters independently is critical to tailoring localized recovery strategies, their long-term survival hinges on restoring connectivity. Management priorities must urgently shift toward establishing, restoring, and maintaining continuous ecological corridors along the major mountain axes [31,32]. This includes strategically modifying or decommissioning non-essential sections of ASF fencing along critical ridges and minimizing human disturbances in transition zones to restore historical gene flow and halt further genetic erosion within this endangered alpine species [29,33]. Nevertheless, we acknowledge that the practical implementation of these designated MUs would benefit from the integration of additional ecological, demographic, and life-history data to provide a more comprehensive baseline for future management decisions [30,31].

## 5. Conclusions

This study successfully implemented a high-resolution 3-RADseq approach to evaluate the genome-wide diversity and spatial genetic architecture of the endangered long-tailed goral (*Naemorhedus caudatus*) in South Korea. By leveraging a robust panel of 9908 high-quality independent SNPs across 34 successfully genotyped individuals, we provided empirical molecular evidence of a profound latitudinal genetic split (ΔK = 2) that sharply segregates the Northern populations from the highly isolated Southern lineage. This pronounced genetic sub-structuring underscores a severe restriction in contemporary gene flow along the Baekdudaegan Mountain Range, indicating that natural topographical barriers and expanding human footprints are likely contributing to genetic isolation within terminal demes.

From a conservation genetics perspective, our findings demonstrate that traditional, single-protected-area management strategies are highly inadequate to guarantee the evolutionary potential and long-term persistence of long-tailed goral. Proactive conservation policies must immediately shift toward establishing, restoring, and maintaining continuous ecological corridors that bridge the fractured Northern and Southern lineages. Mitigating artificial movement barriers—such as extensive African Swine Fever (ASF) mitigation fences and transport infrastructures—and minimizing anthropogenic disturbances in critical transition zones are paramount to restoring historical gene flow, facilitating genetic rescue mechanisms, and enhancing the adaptive resilience of this endangered alpine indicator against future environmental and demographic shocks.

## Figures and Tables

**Table 2:** Mean pairwise *F_ST_* values estimated from 9908 SNPs among five *Naemorhedus caudatus* populations in South Korea.

	BH	HC	UJ	YG	YY
Bonghwa (BH)	0.000				
Hwacheon (HC)	0.172	0.000			
Uljin (UJ)	0.003	0.178	0.000		
Yanggu (YG)	0.138	0.019	0.145	0.000	
Yeongyang (YY)	0.000	0.167	0.022	0.134	0.000

All values were statistically significant at the *p* < 0.01 level.

## Data Availability

Raw DNA sequences obtained from 3-RAD genotyping are available as GenBank accession number SRR38876889.

## Supplementary Materials

The following supporting information can be downloaded at: https://www.mdpi.com/article/10.3390/ani16142189/s1. Table S1: Summary of sequencing statistics, quality control metrics, and bioinformatic filtering steps for the 3-RADseq libraries.

## References

[1] Bragina E., Kim S., Zaumyslova O., Park Y.S., Lee W.S. (2020). *Naemorhedus caudatus*.. The IUCN Red List of Threatened Species.

[2] Jo Y.S., Baccus J.T., Koprowski J.L. (2018). Mammals of Korea.

[3] Jo Y.S., Baccus J.T., Koprowski J.L. (2018). Mammals of Korea: A review of their taxonomy, distribution and conservation status. Zootaxa.

[4] Jo Y.S., Lee H.J., Lee O.S., Park H.B., Cho C.U. (2026). Mapping the distribution and predicting the presence of the Vulnerable long-tailed goral *Naemorhedus caudatus* in South Korea. Oryx.

[5] Frankham R. (2005). Genetics and extinction. Biol. Conserv..

[6] Spielman D., Brook B.W., Frankham R. (2004). Most species are not driven to extinction before genetic factors impact them. Proc. Natl. Acad. Sci. USA.

[7] Manel S., Schwartz M.K., Luikart G., Taberlet P. (2003). Landscape genetics: Combining landscape ecology and population genetics. Trends Ecol. Evol..

[8] Choi S.K., Chun S., An J., Lee M.Y., Kim H.J., Min M.S., Kwon S.W., Choi T.Y., Lee H., Kim K.S. (2015). Genetic diversity and population structure of the long-tailed goral, *Naemorhedus caudatus*, in South Korea. Genes Genet. Syst..

[9] Kim Y.R., Kim H.R., Kim D.H., Jang J.E., Son J.I., Han S.H., Lee H.J. (2025). Phylogenetic status and population genetic structure of the long-tailed goral, *Naemorhedus caudatus* inhabiting the Seoraksan National Park in Korea. Korean J. Environ. Biol..

[10] Lim S., Banjade M., Pandey P., Park Y. (2025). Genetic Structure and Conservation Implications of Long-Tailed Gorals (*Naemorhedus caudatus*) in South Korea: Phylogenetic Analysis and Haplotype Networks. J. For. Environ. Sci..

[11] Allendorf F.W., Hohenlohe P.A., Luikart G. (2010). Genomics and the future of conservation genetics. Nat. Rev. Genet..

[12] Morin P.A., Luikart G., Wayne R.K., The SNP Workshop Group (2004). SNPs in ecology, evolution and conservation. Trends Ecol. Evol..

[13] Narum S.R., Buerkle C.A., Davey J.W., Miller M.R., Hohenlohe P.A. (2013). Genotyping-by-sequencing in ecological and conservation genomics. Mol. Ecol..

[14] Andrews K.R., Good J.M., Miller M.R., Luikart G., Hohenlohe P.A. (2016). Harnessing the power of RADseq for ecological and evolutionary genomics. Nat. Rev. Genet..

[15] Bayona-Vásquez N.J., Glenn T.C., Kieran T.J., Pierson T.W., Hoffberg S.L., Scott P.A., Bentley K.E., Finger J.W., Louha S., Troendle N. (2019). Adapterama III: Quadruple-indexed, double/triple-enzyme RADseq libraries (2RAD/3RAD). PeerJ.

[16] Lee S.R., Choi T.Y., Son D.C. (2024). Multiple introductions of divergent lineages and admixture conferred the high invasiveness in a widespread weed (*Hypochaeris radicata*). Evol. Appl..

[17] Kim H.N., Lee O., Lee H.J., Kim G.C., Kim H.S., Derbridge J.J., Jo Y.S. (2023). The Origin and Invasion Pathway of Brown Rats Rattus norvegicus on Dok-Do Island Revealed by Genome-Wide Markers from 3-RADseq Approach. Animals.

[18] Pritchard J.K., Stephens M., Donnelly P. (2000). Inference of population structure using multilocus genotype data. Genetics.

[19] Evanno G., Regnaut S., Goudet J. (2005). Detecting the number of clusters of individuals using the software STRUCTURE: A simulation study. Mol. Ecol..

[20] Peakall R., Smouse P.E. (2012). GenAlEx 6.5: Genetic analysis in Excel. Population genetic software for teaching and research: An update. Bioinformatics.

[21] Excoffier L., Lischer H.E. (2010). Arlequin suite ver 3.5: A new series of programs to perform population genetics analyses under Linux and Windows. Mol. Ecol. Resour..

[22] Landguth E.L., Cushman S.A., Schwartz M.K., McKelvey K.S., Murphy M., Luikart G. (2010). Quantifying the lag time to detect barriers in landscape genetics. Mol. Ecol..

[23] Shirk A.J., Wallin D.O., Cushman S.A., Rice C.G., Warheit K.I. (2010). Inferring landscape effects on gene flow: A new model selection framework. Mol. Ecol..

[24] Epps C.W., Palsbøll P.J., Wehausen J.D., Roderick G.K., Ramey R.R., McCullough D.R. (2005). Highways block gene flow and cause a rapid decline in genetic diversity of desert bighorn sheep. Ecol. Lett..

[25] Jo Y.S., Gortázar C. (2021). African Swine Fever in wild boar: Assessing interventions in South Korea. Transbound. Emerg. Dis..

[26] Tallmon D.A., Luikart G., Waples R.S. (2004). The alluring simplicity and complex reality of genetic rescue. Trends Ecol. Evol..

[27] Keller L.F., Waller D.M. (2002). Inbreeding effects in wild populations. Trends Ecol. Evol..

[28] Garner A., Rachlow J.L., Hicks J.F. (2005). Patterns of Genetic Diversity and Its Loss in Mammalian Populations. Conserv. Biol..

[29] Frankham R. (2015). Genetic rescue of small inbred populations: Meta-analysis reveals large and consistent benefits of gene flow. Mol. Ecol..

[30] Kim G.C. (2023). Ecology and Conservation of Long-Tailed Goral (*Naemorhedus caudatus*) in South Korea. Ph.D. Thesis.

[31] Hilty J., Worboys G.L., Keeley A., Woodley S., Lausche B., Locke H., Carr M., Pulsford I., Pittock J., White J.W. (2020). Guidelines for Conserving Connectivity through Ecological Networks and Corridors.

[32] Hilty J.A., Lidicker W.Z., Merenlender A.M. (2006). Corridor Ecology: The Science and Practice of Linking Landscapes for Biodiversity Conservation.

[33] Holderegger R., Di Giulio M. (2010). The genetic effects of roads: A review of empirical evidence. Basic Appl. Ecol..

