# Supplementary material for: Population Genetic Structure of the Endangered Long-Tailed Goral (Naemorhedus caudatus) in South Korea Revealed by Genome-Wide Markers from a 3-RADseq Approach
Source: Animals (Basel). 2026 Jul 14;16(14):2189. doi: 10.3390/ani16142189 (PMC13404928; doi:10.3390/ani16142189)
Supplement: Supplementary file 1 [file animals-16-02189-s001.zip › animals-4376287-supplementary.pdf]

| Metric | Value |
|---|---|
| Total sequencing output | 849,646,016 raw reads; 802,406,700 retained (94.4%) |
| Average reads per individual | 22,289,075 ± 20,473,990 (mean ± SD) |
| Coverage depth | 159.1 ± 125.6× (range 23.2–471.3×) |
| Missing data proportion | 0% |

## Loci retained per step

| Step | Loci |
|---|---|
| 1. Retained reads (post-QC) | 802,406,700 |
| 2. Loci assembled | 70,225 ± 24,805 |
| 3. Final loci retained (= SNPs) | 9,908 |
